# Impact of nutrition counseling on anthropometry and dietary intake of multiple sclerosis patients at Kasr Alainy Multiple Sclerosis Unit, Cairo, Egypt 2019–2020: randomized controlled clinical trial

**DOI:** 10.1186/s13690-022-01013-y

**Published:** 2023-01-23

**Authors:** Zeinab Afifi, Amr Hassan, Nebal Abdelrahman, Asmaa El Sayed, Marwa Salem

**Affiliations:** 1 Department of Public Health and Community Medicine, Faculty of Medicine, Cairo University, Cairo, Egypt; 2 Department of Neurology, Faculty of Medicine, Cairo University, Cairo, Egypt; 3 Department of Clinical Nutrition, National Nutrition Institute, Cairo, Egypt

**Keywords:** Multiple sclerosis, Randomized controlled clinical trial, Nutrition counseling, Overweight/obesity, Macronutrients, Micronutrients

## Abstract

**Background:**

Faulty dietary habits and overnutrition are prevalent among Egyptian patients with multiple sclerosis (MS) who do not receive nutrition care as part of treatment. Thus, this study was conducted to identify the effect of nutrition counseling on the nutritional status of patients with MS. This endeavor might provide evidence for the value of counseling in such a setting and advance the integration of nutrition counseling into the routine management of patients with MS.

**Methods:**

A single-blinded, parallel-randomized controlled clinical trial was conducted at Kasr Alainy MS Unit on 120 eligible patients with MS from September 2019 to February 2020. Patients were randomly allocated to either the nutrition counseling intervention group (IG) or the control group (CG). Allocation concealment was performed by using sequentially numbered opaque sealed envelopes. All patients were assessed initially and complied with the Kasr Alainy MS Unit standard management protocol for the study period. Only patients in the IG underwent initial nutrition counseling sessions followed by a monthly evaluation. All patients were assessed at the end of the 3-month follow-up period. Sociodemographic data were gathered through a structured interview. Nutritional status was assessed anthropometrically and via 24-h recall. The 2 groups were compared initially and at the end of the follow-up. Both intention-to-treat and per-protocol analyses were conducted.

**Results:**

At baseline assessment, the prevalence of overweight and obesity was 31.7% and 32.5%, respectively, and the mean body mass index was 27.7 ± 5.7 kg/m^2^. Mean waist circumference was 93.5 ± 11.9 and 99.2 ± 13.1 cm for males and females, respectively. Approximately 27.3% of males and 83.9% of females showed abdominal obesity. After 3 months of counseling, weight, body mass index, waist circumference, nutrient intake and adequacy significantly improved in the IG (*p* < 0.05).

**Conclusion:**

Nutrition counseling significantly improved anthropometric measurements, dietary habits, nutrient intake and adequacy.

**Trial registration:**

The study was registered on ClinicalTrial.gov and was given a code (NCT04217564).

**Supplementary Information:**

The online version contains supplementary material available at 10.1186/s13690-022-01013-y.

## Background

Multiple sclerosis (MS) is the most common non-traumatic disabling disease that affects young adults [1]. MS leads to physical and cognitive disability [2]. According to the MS International Federation, one of every 3,000 individuals worldwide has MS. In Egypt, 9,244 new cases are diagnosed annually (10 new cases/100,000 population/year). Egypt is one of the few countries where the reported prevalence of MS has tripled: from 20/100,000 in 2013 to 59.7/100,000 in 2020. Accordingly, one of every 1,500 Egyptians has MS [3].

Patients with MS were reported to have a double burden of malnutrition: being overweight and obesity occur initially, then weight loss and cachexia occur when disability is advanced [4]. A study conducted in Egypt at Kasr Alainy Multiple Sclerosis Unit (KAMSU) revealed a high prevalence of malnutrition among the patients with relapsing–remitting MS (RRMS) attending the clinic (67.1%). Approximately one-quarter (27.6%) of the studied patients were overweight, 36.8% were obese, and 2.6% were underweight. Unhealthy dietary habits were prevalent among considerable proportions of this group of patients, such as eating junk food, consuming only a few but a large amount at meals, and skipping breakfast [5].

No evidence supports a specific diet for patients with MS but adherence to the dietary guidelines and adopting a healthy lifestyle are suitable choices [6]. Dietary interventions have been claimed to improve the nutritional status of patients with MS [6]. In a clinical setting, nutritional care is provided through nutrition counseling. The goal of nutrition counseling is to produce a desirable change in dietary habits to achieve and maintain healthy body weight, macronutrient balance, and micronutrient adequacy. In this two-way interaction, a client and a trained counselor interpret the results of a nutrition assessment, identify individual nutrition needs and goals, discuss how to fulfill these goals, agree on the next steps, and focus on practical actions to fulfill nutrition needs and the benefits of favorable dietary habits [7].

The aim of this study was to identify the effect of nutrition counseling on the nutritional status of patients with MS to provide evidence for the value of counseling in such a setting and advance the integration of nutrition counseling into the routine management of patients with MS.

## Methods

### Study design, setting, and population

The researchers conducted a single-blinded, parallel-randomized controlled clinical trial (RCT) among patients with MS registered at KAMSU, Faculty of Medicine, Cairo University. The patient flow rate was between 13 and 25 patients per working day.

### Sample size, recruitment of participants, and randomization technique

The sample size was calculated using Statcalc [8]. Assumptions for the calculation of sample size were as follows: a percentage of outcome (bad dietary habits and/or high BMI) in the non-exposed 70% [5]; relative risk, 0.6; alpha, 0.05; and power, 80. The calculated sample size was 100: 50 intervention subjects and 50 control subjects [9]. A 20% dropout rate was assumed; thus, the sample size became 120 patients: 60 intervention and 60 controls.

The sample comprised patients whose records fulfilled the inclusion criteria: diagnosed with RRMS, Secondary Progressive MS (SPMS), or Primary Progressive MS (PPMS); both sexes, aged 20–65 years; and completed secondary school education, its equivalents, or higher education. The exclusion criteria were as follows: clinically isolated syndrome, a relapse, diabetes mellitus, malabsorption syndrome, a food allergy (e.g., to milk and wheat), bariatric surgeries, special diets, drugs that affect appetite (e.g., psychotropic drugs or steroids). And being pregnant or lactating. Eligible patients who consented to participate in the study (120 patients) were randomized by block design into either a control (CG) or an intervention group (IG). The randomization ratio was 1:1. The block size was 4 [10].

**Fig. 1:**
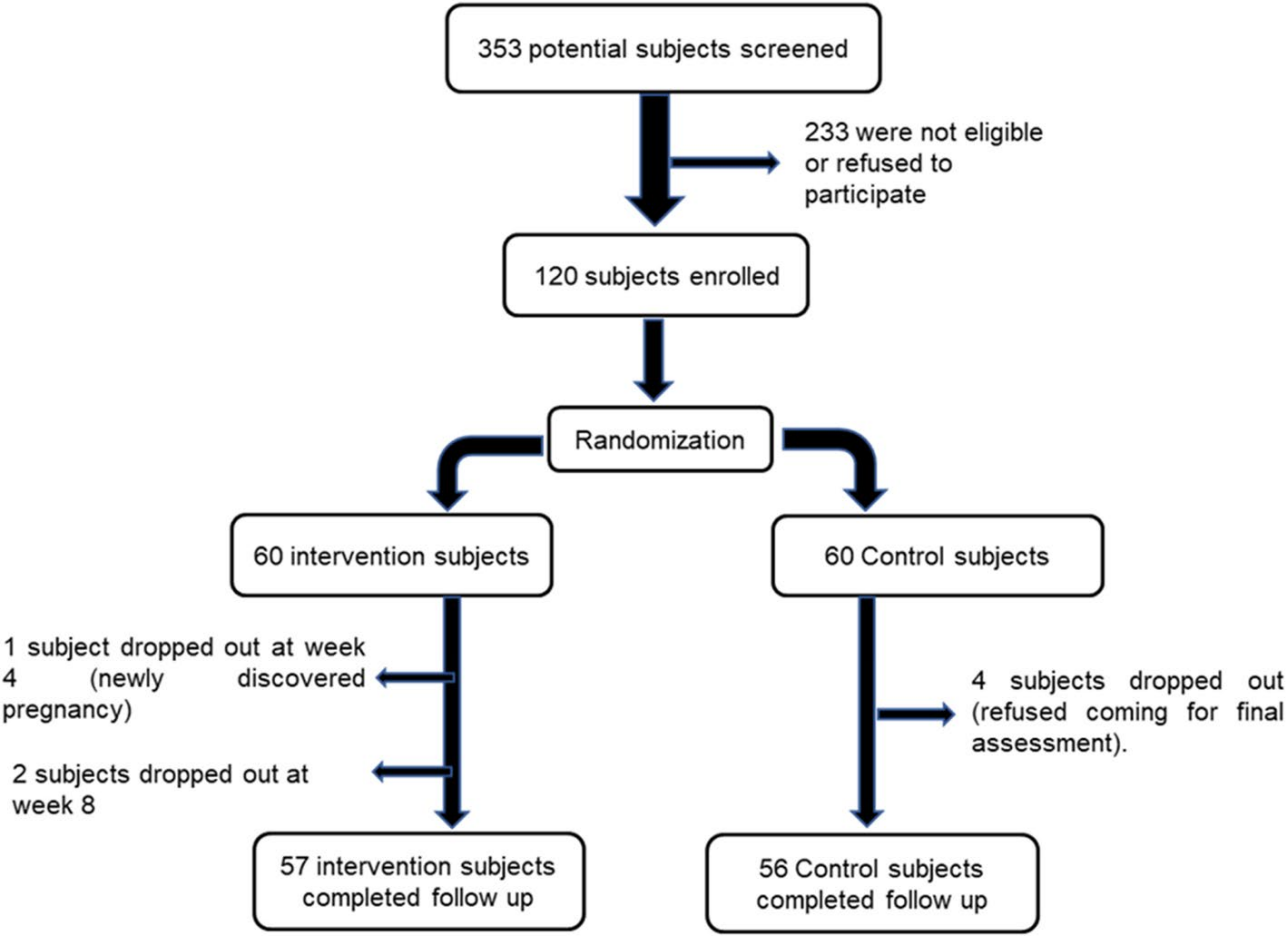
The participants’ flow during the study of the impact of nutrition counseling on patients with multiple sclerosis attending Kasr Alainy Multiple Sclerosis Unit, Cairo, Egypt 2019–2020

The allocation was concealed in sequentially numbered opaque sealed envelopes. Each participant chose a sealed envelope and was assigned to the group specified in that envelope. Figure 1 shows the participants’ flow during the study.

### Study phases

#### Preparatory phase

The researchers prepared a booklet in Arabic with educational material and instructions to be followed by the study participants. The ChooseMyPlate model was adopted and the concept of food groups with healthy choices from each group was illustrated [11]. Foods with positive and negative effects of MS were emphasized [12, 13]. Dietary records were included in the booklet to check patients’ adherence to the instructions. Color-coded sets to measure the quantity of different foods were purchased, and cards to illustrate each bushel in household equivalents were printed.

### Pre-intervention phase (the initial assessment)

#### The following data was gathered through a structured face-to-face interview and/or measurement:

1. Personal, sociodemographic (age, sex, marital status, occupation, education, residency, and telephone/mobile number), and clinical data (disease subtypes, duration, and disability status). Disability was assessed by the Expanded Disability Status Scale (EDSS) score [14].
2. Twenty-four hour dietary recall, detailed data on food preparation, ingredients in mixed dishes, and the brand names of commercial products. The amounts of each food consumed were estimated using standard measuring cups and spoons, as well as common-size containers (e.g., bowls, cups, and glasses) [12]. Daily nutrient intake was analyzed using National Nutrition Institute food composition tables for Egyptian foods [15]**.** Patients were classified by their level of consumption and compared with the FAO, World Health Organization (WHO), and UNU, 2001 human energy requirements [16] and the FAO and WHO 2001 human vitamin and mineral requirements [17]*.* Consumption < 50% was considered unsafe; ≥ 50%–75% was considered unacceptable; >75%–120% was considered acceptable, and > 120% was considered overconsumption [16, 17]*.*
3. Anthropometry. Measurements were collected according to the CDC Anthropometry Procedures Manual [18]. Weight was measured by the German Beurer Glass Bathroom Scale model GS11. Height was measured by the Indian wall-mounted DESCO stature meter (Model HSHA 101) with a resolution of 0.5 cm. Waist circumference was measured using a measuring tape at the end of a normal expiration to the nearest 0.1 cm. BMI was calculated using the standard formula weight (kg)/height (m)^2^. Cutoff points for waist circumference were those set by the WHO [19]. A waist circumference of > 94 cm in males or > 80 cm in females indicated an increased risk of metabolic complications. A waist circumference of > 102 cm in males or in > 88 cm females indicated a substantially increased risk of metabolic complications.

### Intervention (only for the IG)

Each patient was scheduled to receive three sessions 4 weeks apart. The first session was for nutrition counseling. The second and third sessions were to follow-up on patients’ adherence to the nutrition plan and to answer their questions.

- i Each patient was individually counseled. Dietary intake during the last 24 h, daily effort, practice of sports, duration of sedentary habits, sun exposure, and dietary supplement intake were identified. Patient caloric requirements were calculated by the Harris Benedict equation [18]. It was adjusted according to BMI. Patients with acceptable BMI (20–25 kg/m2) [20, 21] who did not report an unintentional weight loss of 5% or more of their body weight in the previous 3–6 months received diet menus that fulfilled their daily caloric requirements to maintain their weights. Patients with a BMI of ≤ 20 kg/m2 and/or patients who reported an unintentional weight loss of 5% or more of their body weight in the previous 3–6 months received diet menus with an extra 500 kcal added to their daily caloric requirements to gain 0.5 kg/week [21, 22]. Overweight and obese patients (BMI ≥ 25 kg/m^2^) received diet menus with a daily caloric restriction of 22% of their daily caloric requirement [23].
- ii A tailored diet menu of a 7-day meal plan to cover all meals and snacks was provided considering the patient’s preferences, working hours, sleeping pattern, and economic class. Preprepared menus were available from the counselor for different caloric intakes (from1000 to 3000 kcal). Those menus were prepared at the National Nutrition Institutes (NNI) and provided Egyptian food items suitable for low and middle socioeconomic classes and included various choices to fulfill participant preferences. During the session, the matching menu with the patient caloric requirements was selected by the counselor and discussed thoroughly with the patient.
- iii At the end of the counseling session, each patient received a package containing an instruction booklet, dietary compliance records, a food and beverage color-coded measuring set, a card illustrating household equivalents of each bushel of the color-coded set, and the tailored diet menu. A WhatsApp group was created. All patients with smartphones and WhatsApp accounts (100% of the patients) were added to the group during the first session. Regular messages were posted by the group’s administrator to motivate patients to maintain a healthy diet and remind them of their follow-up and final assessment appointments.

The CG did not receive nutrition counseling during the study period but was instructed to attend the final assessment. Except for the counseling, the CG received the same care from the clinic's medical staff. After the final assessment, the CG received the nutrition counseling material to gain its expected benefits.

### Post-intervention phase (the final assessment)

The final assessment of all patients in the IG and CG was 3 months after the initial assessment. The same tools were used for the initial and final assessment. The IG was asked about their compliance *(always, often, sometimes, rarely, or never)* with the prescribed regimen (described by the clinic neurologist in addition to the dietary advice). The CG group was also asked about their compliance with the conventional regimen prescribed by the clinic neurologist. Patients who reported their adherence as *always, often, or sometimes* were considered compliant, and patients who reported *rarely or never* were considered non-compliers. The statistician analyzing the data was blinded to the study arms.

### Statistical analysis

Pre-coded data were fed into the computer using Microsoft Office Excel Software 365. Data were transferred to the IBM SPSS version 20 Statistical Package for Social Science Software [24] and the R Statistical Environment (version 4.0.2) for statistical analysis [25].

Categorical variables were expressed in percentages. Quantitative variables were expressed as the mean and standard deviation. The IG and CG were initially compared by their sociodemographic and MS disease characteristics (t test, *Mann–Whitney U,* chi square, and *Fisher’s exact test*). The independent variable was counseling. Outcome variables were quantitative (i.e., weight, BMI, waist circumference, food group intake and nutrient intake) and categorical (i.e., BMI categories, risk of metabolic complications, risk of malnutrition, adequacy of nutrient intake, activity, sun exposure, and supplement intake). Statistical tests used to assess the effect of counseling were ANCOVA in the case of quantitative outcomes, chi-square or Fisher’s exact test in the case of categorical outcomes, and the Mann–Whitney U test in the case of ordinal or continuous but not normally distributed outcomes. Parametric ANCOVA is robust to violations of either normality or homoscedasticity [26].

Three and four patients (5% and 6.7%) of the IG and CG, respectively did not continue the study untill the final assessment. The total dropout rate of the study was 5.8%. All other patients were included in the analysis. This study conducted an intention-to-treat analysis (ITTA) and then a per-protocol analysis (PPA) of the compliers.

## Results

### Sociodemographic and clinical characteristics of the studied patients

Table 1 shows the sociodemographic and clinical characteristics of the enrolled 120 patients at the baseline assessment and compares them in the IG and CG. The mean age of enrolled patients was 33.1 ± 7.5 years; females represented more than 70%. Regarding the group of 120 patients, 41.7% were university graduates; 45% of them received secondary education or equivalent; two-thirds were married; 53.3% were unemployed or housewives; and 75.8% lived in urban communities. All the studied sociodemographic characteristics did not differ significantly between the IG and CG (*P* ≥ 0.05).

**Table 1:** Sociodemographic and disease characteristics of studied patients with multiple sclerosis at Kasr Alainy Multiple Sclerosis Unit, Cairo, Egypt 2019–2020

Characteristics		IG (*N* = 60)	CG (*N* = 60)	Total (*N* = 120)	*P* value #
Age in years *(mean* ± *SD)*		33.5 ± 8.4	32.7 ± 6.4	33.1 ± 7.5	0.659
Gender, N *(%)*	Female	44 (73.3%)	43 (71.7%)	87 (72.5%)	0.838
	Male	16 (26.7%)	17 (28.3%)	33 (27.5%)	
Education, N *(%)*	Secondary	28 (46.7%)	26 (43.3%)	54 (45%)	0.459
	Intermediate institutes	8 (13.3%)	4 (6.7%)	12 (10%)	
	University graduate	23 (38.3%)	27 (45%)	50 (41.7%)	
	Postgraduate degree	1 (1.7%)	3 (5%)	4 (3.3%)	
Occupation, N *(%)*	Non-working/ housewife	29 (48.3%)	35 (58.3%)	64 (53.3%)	0.535
	Unskilled manual worker	3 (5%)	3 (5%)	6 (5%)	
	Skilled manual worker/ Farmer	4 (6.7%)	6 (10%)	10 (8.3%)	
	Trades/ Business	5 (8.3%)	1 (1.7%)	6 (5%)	
	Semi-professional	8 (13.3%)	8 (13.3%)	16 (13.3%)	
	Professional	4 (6.7%)	4 (6.7%)	8 (6.7%)	
	Student	7 (11.7%)	3 (5%)	10 (8.3%)	
Marital Status, N *(%)*	Married	41 (68.3%)	39 (65%)	80 (66.7%)	0.921
	Single	18 (30%)	20 (33.3%)	38 (31. 7%)	
	Divorced	1 (1.7%)	1 (1.7%)	2 (1.7%)	
Residency, N *(%)*	Urban	49 (81.7%)	42 (70%)	91 (75.8%)	0.136
	Rural	11 (18.3%)	18 (30%)	29 (24.2%)	
Disease subtype, N (%)	Relapsing Remitting Multiple Sclerosis (RRMS)	51 (85%)	53 (88.3%)	104 (86.7%)	0.582
	Secondary Progressive Multiple Sclerosis (SPMS)	9 (15%)	6 (10%)	15 (12.5%)	
	Primary Progressive Multiple Sclerosis (PPMS)	0 (0.0%)	1 (1.7%)	1 (0.8%)	
Duration of the disease in years *(mean* ± *SD)*		6.6 ± 6.1	7.3 ± 4.5	7 ± 5.4	0.069
EDSS## *(mean* ± *SD)*		3.4 ± 1.8	2.9 ± 1.5	3.2 ± 1.7	0.091

^*#*^* P value: Mann–Whitney U test for age, duration of the disease, and EDSS; Pearson chi-square test for gender and residency; and Fisher’s Exact test for education, occupation, marital status and disease subtypes*

^##^EDSS: Extended Disability Status Score

RRMS was the major subtype of MS affecting patients in the IG and CG (85% and 88.3%, respectively). One patient in the CG had PPMS. The two groups were comparable regarding MS subtypes and the average disease duration which was 6.6 ± 6.1 and 7.3 ± 4.5 years for the IG and CG, respectively (*P* ≥ 0.05). They were also comparable in disability status. The EDSS was 3.4 ± 1.8 and 2.9 ± 1.5 in the IG and CG, respectively (*P* = 0.091). Medical characteristics did not differ significantly between the IG and CG (*P* ≥ 0.05).

### Baseline nutritional status of the studied patients

At the baseline assessment, the prevalence of overweight and obesity was 31.7% and 32.5%, respectively. The mean BMI was 27.7 ± 5.7 kg/m^2^. Approximately 27.3% of males and 83.9% of females were at substantially increased risk of metabolic complications due to abdominal obesity. The mean baseline waist circumference was 93.5 ± 11.9 and 99.2 ± 13.1 cm for males and females, respectively. One-quarter or more of the participants overconsumed energy, carbohydrates, fats and proteins (25%, 25.8%, 31.7%, and 78.3%, respectively). After randomization, the IG and CG were comparable in sociodemographic and disease characteristics and in almost all outcome variables (anthropometry, nutritional status, nutrient intake, intake of supplements, sun exposure and activity), *P* > 0.05. The two groups differed significantly in their intake of added fat.

### ITTA before and after intervention

#### Anthropometric assessment

ITTA showed that after the intervention, mean weight, BMI and waist circumference significantly decreased in counseled females (*P* = 0.000, 0.001, and 0.011 respectively) but not in counseled males. The mean waist circumference of females decreased from 101.5 ± 13.5 cm to 100.7 ± 11.5 cm in the IG and increased from 97.1 ± 12.3 cm to 97.9 ± 12.3 cm (*P* = 0.011) in the CG (Table 2). Neither MS duration nor EDSS altered the effect of the intervention on the three anthropometric measurements. Inclusion of MS subtype did not alter the effect of intervention on weight and BMI but it did modify the significance of its effect on waist circumference (*P* = 0.065 instead of 0.005).

**Table 2:** Mean of anthropometric measures of the studied patients with multiple sclerosis at Kasr Alainy Multiple Sclerosis Unit, Cairo, Egypt 2019–2020

Anthropometry	Pre-intervention				Post-intervention				*P* value #
	**IG (*N* = 60)**		**CG (*N* = 60)**		**IG (*N* = 57)**		**CG (*N* = 56)**		
	**Mean**	**SD**	**Mean**	**SD**	**Mean**	**SD**	**Mean**	**SD**	
**Weight (kg)**									
- Female	76.4	17.6	73.7	15.3	74.9	16.4	74.5	15.7	0.000*
- Male	78.4	16.3	69.3	11.2	77.0	14.6	68.9	11.5	0.652
**BMI (kg/m2)**									
- Female	29.9	6.2	27.7	5.4	29.2	5.6	28.0	5..6	0.001*
- Male	25.3	4.4	24.5	4.1	25.0	3.9	24.3	4.3	0.455
**Waist circumference (cm)##**									
- Female	101.5	13.5	97.1	12.3	100.7	11.5	97.9	12.3	0.011*
- Male	95.3	13.8	91.8	10.1	93.8	12.1	91.4	10.5	0.280

^*^Significant P value

^*#*^*P value: ANCOVA*

^*##*^*Normal value for males and females is below 94 cm and 80 cm, respectively*

Weight loss expressed as the percent of initial weight was significantly higher in the counseled overweight and obese patients (3.9 ± 4.3% and 4.5 ± 4.6% of their initial weights, respectively). In the CG, after the 3-month follow-up, overweight patients lost less weight (0.24% ±2.3% of their initial weights), and obese patients gained more weight (0.32% ± 1.1% of their initial weights). The observed changes were statistically significant for overweight and obese patients (*P* = 0.001 and 0.012, respectively). After counseling, the percentage of obese patients decreased in the IG group from 40% to 31.6% after counseling (*P* = 0.025).

### Dietary assessment

**Fig. 2:**
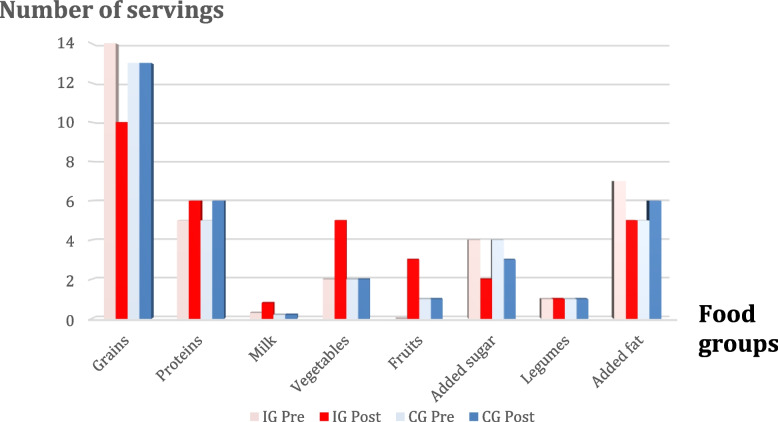
Number of servings from food groups before and after counseling in intervention group (IG) and control group (CG) of studied patients with multiple sclerosis, Kasr Alainy Multiple Sclerosis Unit, Cairo, Egypt 2019–2020

The analysis of 24-h recall showed that the two groups initially consumed a comparable number of servings during the last 24 h from all food groups (*P* ≥ 0.05). However, for added fat, the IG reported consuming more than that reported by the CG did (7 ± 3 versus 5 ± 2 respectively). After counseling, the IG changed their intake from food groups favorably. Their consumption significantly decreased as follows: grains (13.7 ± 6.7 to 10.4 ± 4.0 serving/day), added sugar (4.1 ± 3.1 to 1.7 ± 1.7 serving/day) and added fat (6.6 ± 3.5 to 4.5 ± 3.5 serving/day). Their consumption increased as follows: milk (0.3 ± 0.5 to 0.8 ± 0.8 serving/day), vegetables (2.0 ± 1.8 to 4.8 ± 2.6 serving/day), and fruits (0.5 ± 0.9 to 2.6 ± 1.5 serving/day) (Fig. 2). The counseling intervention significantly increased the number of servings consumed from milk (*P* = 0.000), vegetables (*P* = 0.000), and fruits (*P* = 0.000) and significantly reduced the number of servings consumed from grains (*P* = 0.002), added sugar (*P* = 0.000), and fats (*P* = 0.000).

Table 3 shows the mean 24-h intake of energy, macronutrients, and some micronutrients before and after counseling. Nutrition counseling significantly improved the intake of most nutrients (energy, fat, Na, P, fiber, K. Mg and vitamin C**)**. The IG patients consumed significantly less energy, protein, fat, carbohydrate, sodium, phosphorus and zinc. The IG consumed significantly more fibers, potassium, magnesium, and vitamins A and C. The observed effect of counseling on all nutrients’ intake remained to be the same after including MS type as a factor and the duration of disease and EDSS as covariates**.** Considering the adequacy of intake after counseling, Table 4 shows that two-thirds (66.7%) of the IG and 39.3% of the CG consumed acceptable amounts of calories. Fewer IG patients overconsumed calories than the CG patients did (8.8% vs. 26.8%, respectively, *P* = 0.015). Nearly two-thirds (64.9%) of the IG and 30.4% of the CG patients consumed an acceptable amount of fat, and more members of the CG overconsumed fat than IG (8.8% and 26.8%, respectively, *P* = 0.002). A similar but marginally significant effect was observed in the case of carbohydrates (*P* = 0.051). Similarly, counseling had favorable impact on the acceptable consumption of magnesium; iron; and vitamins C, B1 and B2 but not on calcium; zinc, and vitamin A.

**Table 3:** Mean 24-h intake of energy, macronutrients and some micronutrients of the studied patients with multiple sclerosis at Kasr Alainy Multiple Sclerosis Unit, Cairo, Egypt 2019–2020

**Intake**	**Pre-intervention**				**Post-intervention**				***P* value #**
	**IG (*N*** **= 60)**		**CG (*N* = 60)**		**IG (*N* = 57)**		**CG (*N* = 56)**		
	**Mean**	**SD**	**Mean**	**SD**	**Mean**	**SD**	**Mean**	**SD**	
Energy (Kcal)	2528.8	1008.3	2413.7	889.4	2130.1	728.8	2517.7	924	0.004*
Protein (grams)	89.3	35.8	84.6	37.9	72.3	27.1	93.8	45	0.001*
Fat (grams)	88.9	42.4	78	36	68.4	32.5	84.9	37.5	0.008*
Carbohydrates (grams)	342.4	150	341.6	128.9	300.4	96.6	342.1	147	0.053
Fibers (grams)	8.6	5.2	8.8	3.9	21	8.3	9.3	4.6	0.000*
Na (mg)	3222.7	1450.7	3274.8	1488.5	2067.1	1383	3504.9	2012.1	0.000*
K (mg)	2380.8	1205	2339.4	819.2	3044.3	838.6	2604.5	1032.3	0.015*
Mg (mg)	127.4	74.3	133.2	69.7	160.4	48	132.8	65	0.008*
Ca (mg)	694.3	394.6	729.2	376.2	695.6	254.9	720	371.1	0.813
P (mg)	1190.6	541.5	1304.7	602.8	1093.1	354.6	1328.8	668.4	0.04*
Zn (mg)	12.9	7	12	5.2	9.9	5	13.2	7.8	0.005*
Fe (mg)	16.8	9.6	16.3	7.1	16.9	5.8	18.4	9	0.261
Cu (mg)	1	0.7	0.9	0.5	1	0.3	1	0.7	0.749
Vit. A (μg)	430	1056.5	628.4	840.3	1370.8	1470	1025.1	1701.5	0.254
Vit. C (mg)	43.7	133.2	44.8	38.9	96.3	57.1	36.8	31.6	0.000*
Vit. B1 (Thiamin) (mg)	0.9	0.6	0.9	0.4	0.9	0.3	1	0.5	0.835
Vit. B2 (mg)	1	1	1	0.9	1.1	0.6	1.2	2	0.558

^#^ *P* value: ANCOVA

^*^Significant *P* value

**Table 4:** Distribution of studied patients with multiple sclerosis at Kasr Alainy Multiple Sclerosis Unit, Cairo, Egypt 2019–2020 according to adequacy of intake of energy and macronutrients among the study groups pre- and post-intervention

Compared intake with RDA^##^		Pre-intervention				Post-intervention				*P* value #
		**IG (*N* = 60)**		**CG (*N* = 60)**		**IG (*N* = 57)**		**CG (*N* = 56)**		
		**N**	**%**	**N**	**%**	**N**	**%**	**N**	**%**	
Calories	*Unsafe*	4	6.7	8	13.3	4	7.0	4	7.1	0.015*
	*Unacceptable*	14	23.3	11	18.3	10	17.5	15	26.8	
	*Acceptable*	26	43.3	27	45.0	38	66.7	22	39.3	
	*Overconsumption*	16	26.7	14	23.3	5	8.8	15	26.8	
Carbohydrate	*Unsafe*	11	18.3	10	16.7	7	12.3	11	19.6	0.051
	*Unacceptable*	17	28.3	10	16.7	10	17.5	10	17.9	
	*Acceptable*	15	25.0	26	43.3	36	63.2	23	41.1	
	*Overconsumption*	17	28.3	14	23.3	4	7.0	12	21.4	
Fat	*Unsafe*	8	13.3	10	16.7	6	10.5	8	14.3	0.002*
	*Unacceptable*	9	15.0	12	20.0	8	14.0	12	21.4	
	*Acceptable*	22	36.7	21	35.0	37	64.9	17	30.4	
	*Overconsumption*	21	35.0	17	28.3	6	10.5	19	33.9	
Protein	*Unsafe*	1	1.7	1	1.7	0	0.0	0	0.0	0.091
	*Unacceptable*	3	5.0	1	1.7	0	0.0	4	7.1	
	*Acceptable*	6	10.0	14	23.3	10	17.5	6	10.7	
	*Overconsumption*	50	83.3	44	73.3	47	82.5	46	82.1	

^*#*^* P value: Pearson chi-square test for carbohydrate and fat, Fisher’s Exact test for calories and proteins*

^*##*^*Unsafe* < *50%, Unacceptable 5050—75%, Acceptable 7575—120%, Overconsumption* ≥ *120% of RDA*

^*^Significant P value

After counseling, the IG group spent a shorter time in sedentary activity than the CG (4.7 ± 2.2 h/day versus 5.9 ± 2.5 h/day). Table 5 shows that after counseling, more members of the IG (70.2%) practiced sports than those in the CG did (18%); 63.2% and 62.5% of the IG and CG patients were taking vitamin and mineral supplements, one-third of the IG patient were exposed to the sun vs. 7.1% in the CG. The observed impact of counseling on the duration of sedentary time, sports practice and exposure to the sun was statistically significant. Counseling significantly reduced sedentary time (*P* = 0.000), increased the practice of sports (*P* = 0.000) and exposure to the sun (*P* = 0.000), but did not significantly affect vitamin and mineral supplement intake (*P* = 0.942**)**.

**Table 5:** Distribution of the studied patients with multiple sclerosis at Kasr Alainy Multiple Sclerosis Unit, Cairo, Egypt 2019–2020 by activity, vitamin and mineral supplement intake, exposure to the sun and risk of malnutrition before and after counseling

**Physical activity**		**Pre-intervention**				**Post-intervention**				***P* value #**
		**IG (N = 60)**		**CG (N = 60)**		**IG (N = 57)**		**CG (N = 56)**		
		**N**	**%**	**N**	**%**	**N**	**%**	**N**	**%**	
**Daily Effort**	Very light	21	35.0	18	30.0	13	22.8	23	41	0.111
	Light	31	51.7	31	51.7	33	57.9	24	42.9	
	Moderate	8	13.3	11	18.3	11	19.3	9	16.1	
**Sport Practice**	≥ 3times/Week	3	5	8	13.3	23	40.4	5	9	0.000*
	< 3times/Week	12	20	4	6.7	17	29.8	5	9	
	No	45	75	48	80	17	29.8	46	82	
**Supplement intake**	Yes	36	60	42	70	36	63.2	35	62.5	0.942
	No	24	40	18	30	21	36.8	21	37.5	
**Exposure to sun**	Yes	14	23.3	7	11.7	19	33.3	4	7.1	0.000*
	No	46	76.7	53	88.3	38	66.7	52	92.9	

^#^ *P*-value Pearson chi-square or Fisher’s Exact test

^*^Significant *P* value

### PPA of the compliers

**T**he results obtained by PPA were consistent with those obtained from ITTA (Refer to supplementary files).

## Discussion

This study revealed a high prevalence of overweight, obesity and central obesity among the enrolled 120 patients, close to that reported for patients with MS in the same unit in a prior study [5]. The prevalence of overweight and obesity is higher than that reported by Marck et al. [27]. Mean waist circumference is also higher than that reported by Drehmer et al. [28], indicating a higher risk of metabolic complications than the other finding [19]. The observed higher prevalence in this study than in theirs can be attributed to the overconsumption of macronutrients, low intake of dietary fibers and the sedentary nature of most of the patients. The mean 24-h energy, carbohydrates, proteins, and fat consumption were higher in our study than that reported by Armon-Omer et al. [29] and the lower fiber intake accounts for the difference in mean BMI between the two studies (27.7 ± 5.7 kg/m^2^ and 25.0 ± 4.4 kg/m2, respectively).

The current trial revealed that counseling improved the IG anthropometry, food choices and most nutrients’ intake and adequacy compared to the CG. Counseling significantly increased the practice of sports, sun exposure and reduced the time spent on sedentary activities.

The effect of the intervention on weight, BMI, and waist circumference differed in the two sex groups, indicating an intervention–sex interaction. Nutrition counseling significantly helped female patients with MS improve their weight, BMI, and waist circumference. It did not significantly affect the three measures in males. An explanation for the differential effect is that the number of males was less than half that of females (33 and 87, respectively); moreover, males had lower mean BMI and waist circumference than females (*P* < 0.05) did because most of men (60.6%) started at a normal weight; approximately one-third of males had the more severe forms of disease (SPMS and PPMS), and that was 6.9% for females (*P* = 0.002); they had earlier onset of disease, longer duration, and higher EDSS (*P* < 0.05) (Refer to supplementary files). Inclusion of disease factors in the analysis revealed marginally significant interaction between intervention and MS subtype in males but not in females (*P* = 0.056).

The PPA showed that more weight was lost by compliant overweight (*P* = 0.000) and obese patients (*P* = 0.000) after the excluding non-compliers who diluted the change observed in the ITTA. Additionally, the PPA highlighted the significant change in the compliant male’s waist circumference (*P* = 0.043) in contrast with the insignificant change revealed by the ITTA. (*P* = 0.280) (Refer to supplementary files). The link between the nutritional status and the clinical condition of this group requires further research. A few findings have revealed that overweight and obesity impair the physical and mental health of patients with MS [30]. Moreover, high BMI and waist circumference in patients with MS may exacerbate disease symptoms and the accumulation of disabilities [31, 32].

Per the pre-intervention assessment, dietary fiber was consumed at less than the acceptable levels (8.7 ± 4.6 g/day in all enrolled patients, 8.0 ± 4.1 in females and 10.6 ± 5.2 in males). Nutrition counselling induced significant improvement in fiber intake in the IG from 8.6 ± 5.2 g to 21.0 ± 8.3 g (*P* = 0.000), and the level of intake of males (27.0 g) and females (20.9 g) became close to the minimum recommended amount compared with the 2020–2025 Dietary Guidelines (22–28 g for women and 28–34 g for men over the age of 19) [33]. Thus, the emphasis on eating fiber-rich foods should increase. High-fiber and low-caloric dietary intake may reduce disease severity and suppress inflammatory conditions in patients with MS [34]. Furthermore, energy intake and dietary fibers are good predictors for EDSS and anthropometric indices (BMI and percentage of body fat) [34].

Regarding micronutrients, the intake of sodium, potassium, calcium, phosphorous, magnesium, iron, zinc and copper and vitamins A, C, B1, and B2 intake was comparable in the IG and CG in the pre-counseling assessment (*P* > 0.05). Those intakes were lower than the RDA for all nutrients except sodium, which was much higher than the RDA, and phosphorous and copper which were slightly higher than the RDA [17]. The lower intake of calcium, magnesium, zinc, and iron was consistent with finding in Armon-Omer et al. [29].

Counseling also improved micronutrient intake, ANCOVA analysis revealed a significant increase in the consumption of potassium, magnesium, and vitamin C and a decrease in that of sodium, phosphorus, and zinc (*P* < 0.05). Impairments in micronutrient intake might be of clinical significance in MS because they contribute to existing symptoms, such as muscle wasting, weakness, fatigue, and muscle spasms [20, 35]. The observed effect is attributed to the improved dietary habits, decreased sedentary hours, and increased physical activity. The IG significantly increased their intake of milk, vegetables and fruits and decreased their intake of grains, added sugar and fat (*P* < 0.05). Milk is an excellent source of many vitamins and minerals, including vitamin B12, calcium, riboflavin, and phosphorus. Milk is often fortified with other vitamins, especially vitamin D [36]. Vegetables and fruits are good sources of vitamins and minerals including vitamin C, carotene, calcium, magnesium, iron, and potassium [37]. Minerals homeostasis plays a fundamental role in the regulation of the CNS functions. In addition, mineral deficiencies have been found in the serum of patients with MS, namely, iron, magnesium, and zinc [35, 38, 39]. These imbalances have been linked to demyelination, perhaps involving oxidative stress [40]. Lower serum zinc levels in patients with MS are commonly observed. Zinc plays a critical role in modifying neuronal excitability and synaptic plasticity [39, 41].

Sodium was consumed in very large amounts by the IG and CG (3222.7 mg and 3274.8 mg/day, respectively). This consumption was higher than the 2300 mg recommended by the 2020–2025 Dietary Guidelines for individuals aged older than 19 years [33]. It was also higher than the mean sodium intake reported in the Armon-Omer et al. (2392.66 mg) [29]. Kleinewietfeld suggested increased dietary salt intake as an environmental risk factor for the development of autoimmune diseases by inducing pathogenic cells and related proinflammatory cytokines [42]. Those cells have been shown to be involved in the development of MS [43]**.** Although the evidence linking high salt intake and MS is contradictory, combining the DASH diet with low sodium intake might benefit individuals with MS. It also improves the vascular health of patients with MS [6].

## Conclusion

Nutrition counseling significantly improved anthropometric measurements, dietary habits, and nutrient intake and adequacy among the studied sample of patients with MS attending KAMSU. Accordingly, nutrition counseling and care must be considered an integral part of the health care of patients with MS at KAMSU and elsewhere.

### Study strengths and limitations

To identify the effect of nutrition counseling on the nutritional status of patients at KAMSU, we conducted a RCT, the gold standard for measuring the effectiveness of a new intervention or treatment. This study’s findings should be interpreted while considering the following limitations: blinding of patients and health care providers was not possible owing to the nature of intervention; however, we blinded the statistician who analyzed the data. Nutrition counseling was provided by a specialized nutritionist, and follow-up was optimized through phone calls and WhatsApp messaging. Forty-five patients in the IG and 50 patients in the CG (78.9% and 89.3%, respectively) were compliant with the prescribed regimens, as reported in the final assessment. Additionally, the 24-h dietary recall may have some limitations, such as recall bias, inaccurate estimation of portion sizes, possible over/under-reporting of certain foods, and that data from a single day cannot accurately represent the respondent’s usual intake. One of the researchers, an ESPEN-qualified clinical nutrition specialist, conducted all interviews to ensure good data quality. Moreover, the 24-h dietary recall was complemented by other tools of nutritional assessment, such as anthropometric and clinical.

### Recommendations

Further studies are recommended to explore the effect of nutrition counseling on MS nutrition status. Those studies may include other parameters such as vitamin D status, lipid profile, blood glucose and HbA1c levels.

## Supplementary information

**Additional file 1: Table S1.** Significant sex differences in disease characteristics and weight categories of studied patients with multiple sclerosis at Kasr Alainy Multiple Sclerosis Unit, Cairo, Egypt 2019-2020. **Table S2.** Mean of weight, Body mass index (BMI) and waist circumference of the compliant studied patients with multiple sclerosis at Kasr Alainy Multiple Sclerosis Unit , Cairo, Egypt 2019-2020. **Table S3. **Weight change of compliant studied patients with multiple sclerosis at Kasr Alainy Multiple Sclerosis Unit, Cairo, Egypt 2019-2020.

## Data Availability

The datasets used and/or analyzed during this study are available from the corresponding author upon reasonable request.

## Abbreviations

ANCOVA: Analysis of covariance adjusted for baseline measures
BMI: Body Mass Index
CDC: Centers for Disease Control and Prevention
CG: Control group
CNS: Central nervous system
DASH: Dietary approaches to stop hypertension
EDSS: Expanded Disability Status Scale
ESPEN: The European Society for enteral and parenteral nutrition
FAO: The Food and Agriculture Organization
IG: Intervention group
ITTA: Intention to treat analysis
KAMSU: Kasr Alainy Multiple Sclerosis Unit
MS: Multiple sclerosis
NNI: National Nutrition Institute
PPA: Per-protocol analysis
PPMS: Primary progressive MS
RCT: Randomized Controlled Trial
RDA: Recommended Dietary Allowance
RRMS: Relapsing–Remitting Multiple Sclerosis
SPMS: Secondary Progressive Multiple Sclerosis
SPSS: Statistical Package of Social Science Software program
UNU: United Nations University
WHO: World Health Organization
