# Supplementary material for: Impact of nutrition counseling on anthropometry and dietary intake of multiple sclerosis patients at Kasr Alainy Multiple Sclerosis Unit, Cairo, Egypt 2019–2020: randomized controlled clinical trial
Source: Arch Public Health. 2023 Jan 23;81:11. doi: 10.1186/s13690-022-01013-y (PMC9869589; doi:10.1186/s13690-022-01013-y)
Supplement: Supplementary file 1 — Additional file 1: Table S1. Significant sex differences in disease characteristics and weight categories of studied patients with multiple sclerosis at Kasr Alainy Multiple Sclerosis Unit, Cairo, Egypt 2019-2020. Table S2. Mean of weight, Body mass index (BMI) and waist circumference of the compliant studied patients with multiple sclerosis at Kasr Alainy Multiple Sclerosis Unit , Cairo, Egypt 2019-2020. Table S3. Weight change of compliant studied patients with multiple sclerosis at Kasr Alainy Multiple Sclerosis Unit, Cairo, Egypt 2019-2020. [file 13690_2022_1013_MOESM1_ESM.docx]

**Supplementary files:**

**Sex differences in disease characteristics and weight categories:**

**Table S1: Significant sex differences in disease characteristics and weight categories of studied patients with multiple sclerosis at Kasr Alainy Multiple Sclerosis Unit, Cairo, Egypt 2019-2020**

| **Characteristics** | | **Females (**87**)** | **Males (33)** | **Total (120)** | **P value** |
| --- | --- | --- | --- | --- | --- |
| Onset of disease *(mean ± SD)* | | 2013.8 ± 4.6 | 2011.1 ± 6.8 | 2013.1 ± 5.4 | 0.014* |
| Duration of the disease in years *(mean ± SD)* | | 6.25 ± 4.6 | 8.9 ± 6.8 | 7.0 ± 5.4 | 0.014* |
| EDSS *(mean ± SD)* | | 2.82 ± 1.6 | 4.12 ± 1.6 | 3.18 ± 1.7 | 0.000* |
| **Disease subtype, N (%)** | Relapsing Remitting Multiple Sclerosis (RRMS) | 81 (93.1%) | 23 (69.7%) | 104 (86.7%) | 0.002* |
|  | Secondary Progressive Multiple Sclerosis (SPMS) | 6 (6.9%) | 9 (27.3%) | 15 (12.5%) |  |
|  | Primary Progressive Multiple Sclerosis (PPMS) | 0 (0.0%) | 1 (1.3%) | 1 (0.8%) |  |
|  | Total | 87 (100%) | 33 (100%) | 120 (100%) |  |
| **BMI# categories, N (%)** | Normal weight | 22 (25.3%) | 20 (60.6%) | 42 (35.0%) | 0.002* |
|  | Obese | 35 (40.2%) | 4 (12.1%) | 39 (32.5%) |  |
|  | Overweight | 29 (33.3%) | 9 (27.3%) | 38 (31.7%) |  |
|  | Underweight | 1 (1.1 %) | 0 (0.0 %) | 1 (0.8 %) |  |
|  | Total | 87 (100.0%) | 33 (100.0%) | 120 (100.0%) |  |

*#BMI: Body mass index*

**Significant P value*

**PPA for anthropometric measurements of the compliers of the two groups (IG and CG)**

Among the enrolled participants, 78.9% and 89.3% of IG and CG patients were compliant to the prescribed regimens as reported in the final assessment (p=0.213). The CG patients were compliant to the conventional regimen of Kasr Al-Ainy multiple sclerosis unit while the IG patients were compliant to the dietary instruction in addition to the conventional regimen of the unit.

Table S2 shows that following the intervention, the mean weight of the compliant patients significantly decreased from 75.1±15.97 kg to 72±14.1kg kg in the IG while it remained almost the same in the control group (72.4±14.6 and 72.3±15.1 kg). Similarly, the mean BMI significantly decreased after the intervention from 28.1±5.8 to 26±4.99 kg/m2 while it remained almost the same in the control group (26.8±5.4 and 26.7±5.6 kg/m2). Counseling intervention significantly reduced weight (p value =0.000) and BMI (p value =0.000).

In addition, the mean waist circumference of females decreased from 101±14 cm to 98.2±11.4 cm in the IG while it remained almost the same in the CG (97.5±12.6 cm and 97.5±13 cm). Regarding males, the mean waist circumference decreased from 96.4±11.5 cm to 94.2±9.7 cm in the IG while it remained almost the same in the CG (92±10.3 cm and 91.6±10.7 cm). There was significant difference in the means waist circumference of female (p= 0.000) and male (p= 0.043) subjects between the IG and the CG in the post-intervention assessment, whilst adjusting for the pre-intervention waist circumference.

**Table S2: Mean of weight, Body mass index (BMI) and waist circumference of the compliant studied patients with multiple sclerosis at Kasr Alainy Multiple Sclerosis Unit , Cairo, Egypt 2019-2020**

| **Anthropometry** | **Pre-intervention** | | | | **Post-intervention** | | | | **P value#** |
| --- | --- | --- | --- | --- | --- | --- | --- | --- | --- |
|  | **IG (N=45)** | | **CG (N=50)** | | **IG (N=45)** | | **CG (N=50)** | |  |
|  | **Mean** | **SD** | **Mean** | **SD** | **Mean** | **SD** | **Mean** | **SD** |  |
| Weight (kg) | 75.1 | 15.97 | 72.4 | 14.6 | 72 | 14.1 | 72.3 | 15.1 | 0.000* |
| BMI (kg/m^2^) | 28.1 | 5.8 | 26.8 | 5.4 | 26.9 | 4.99 | 26.7 | 5.6 | 0.000* |
| Waist circumference (cm)## | | | | | | | | | |
| Female | 101 | 14 | 97.5 | 12.6 | 98.2 | 11.4 | 97.5 | 13 | 0.000* |
| Male | 96.4 | 11.5 | 92 | 10.3 | 94.2 | 9.7 | 91.6 | 10.7 | 0.043* |

*# P value: ANCOVA*

*##Normal value for males is below 94 cm and for females is below 80 cm*

**Significant P value*

Table S3 summarizes the means of the initial weight and the percentages of weight changes of the studied compliant patients after the 3 months follow up. Overweight and obese patients of the IG lost 5.4±2.2 % and 7.4±2.1% of their initial weights respectively while overweight and obese patients of the CG gained more weight (0.3±2.5 % and 0.4±1.2 % of their initial weights) after the 3 months follow up.

**Table S3: Weight change of compliant studied patients with multiple sclerosis at Kasr Alainy Multiple Sclerosis Unit, Cairo, Egypt 2019-2020**

| **Initial BMI# Categories** | **IG (N=45)** | | | **CG (N=50)** | | | **P value##** |
| --- | --- | --- | --- | --- | --- | --- | --- |
|  | **N** | **Initial Weight (kg)** | **% Weight change after 3 months** | **N** | **Initial Weight (kg)** | **% Weight change after 3 months** |  |
|  |  | **Mean (SD)** | **Mean (SD)** |  | **Mean (SD)** | **Mean (SD)** |  |
| **Underweight** | - | - | - | 1 | - | - | - |
| **Normal weight** | 17 | 59.9 (9.8) | 0.68 (4.5) | 18 | 60.3 (6.7) | -1.4 (2.5) | 0.248 |
| **Overweight** | 12 | 78.1 (10.1) | -5.4 (2.2) | 19 | 73.7 (7.8) | 0.35 (2.5) | 0.000* |
| **Obese** | 16 | 90.1 (11.8) | -7.4 (2.1) | 12 | 90.6 (11.6) | 0.35 (1.2) | 0.000* |

^#^BMI: Body Mass Index

^##^ P value: *Mann-Whitney U test*

**Significant P value*
